# Comparative Analysis of Axial Length Measurements by Optical Biometers Based on Partial Coherence Interferometry Versus Optical Low-Coherence Interferometry: An Office Audit

**DOI:** 10.7759/cureus.21883

**Published:** 2022-02-03

**Authors:** Harleen Bhullar, Leland Dhurjon, Christopher Francis, Vishaal Bhambhwani

**Affiliations:** 1 Medicine, American University of the Caribbean School of Medicine, Cupecoy, SXM; 2 Department of Surgery, Thunder Bay Regional Health Sciences Centre, Thunder Bay, CAN; 3 Department of Surgery, Northern Ontario School of Medicine, Thunder Bay, CAN

**Keywords:** cataract, optical low coherence interferometry, partial coherence interferometry, optical biometers, axial length, biometry

## Abstract

Purpose

In this study, we aimed to compare axial length (AL) measurements of the IOLMaster 500 (Carl Zeiss Meditec AG, Jena, Germany), based on partial coherence interferometry (PCI) versus the Aladdin (Topcon Healthcare, Oakland, NJ), based on optical low-coherence interferometry (OLCI), in a clinical setting.

Methods

A retrospective analysis of the records of patients presenting for cataract surgery at an ophthalmology practice between October 2019 and March 2020 was performed. All patients had biometry measurements on the IOLMaster 500 and the Aladdin. Data collected included patient demographics, cataract morphology and type, and AL measurements. The IOLMaster 500 and Aladdin measurements were compared using the unpaired t-test and Chi-squared test.

Results

In total, 393 eyes (197 patients) were included (91 males, 107 females) in the study. The IOLMaster 500 was unable to successfully obtain AL measurements in seven eyes (1.8%) and the Aladdin in 26 eyes (6.6%). The difference was statistically significant (p=0.0007). Advanced and central posterior subcapsular cataracts were common in eyes that had unsuccessful measurements. In the eyes successfully measured, the mean AL for the IOLMaster was 24.04 ±1.32 mm, while it was 24.04 ±1.34 mm for the Aladdin. However, this difference was not statistically significant (p=0.9165).

Conclusion

The IOLMaster 500 performed better in terms of the number of eyes for which AL measurements were successfully obtained compared to the Aladdin. This may be partly related to high volumes of advanced cataracts treated at our practice. However, this being a retrospective study, a cause-and-effect relationship could not be established.

## Introduction

Cataract surgery with the implantation of an intraocular lens (IOL) is one of the most commonly performed ophthalmic surgical procedures [1,2]. To optimize the accuracy of predicting postoperative refraction, formulas have been developed to calculate IOL power on the basis of preoperative measurement of intraocular distances, especially axial length (AL) and keratometry [3,4]. Previously, applanation and immersion ultrasound (contact methods) were used for AL measurements [5]. Later on, optical (non-contact) biometry techniques were developed [6].

Until 2009, the IOLMaster (Carl Zeiss Meditec AG, Jena, Germany), based on partial coherence interferometry (PCI) with a 780 nm diode laser, had been the only device to measure AL by the optical method [7]. Since then, newer instruments have been introduced. One of the newer biometers is the Aladdin (Topcon Healthcare, Oakland, NJ), based on optical low-coherence interferometry (OLCI) using an 830 nm super-luminescent diode [7,8].

A few studies comparing the IOLMaster with the Aladdin are available in the literature [7-10]; however, most were performed in a controlled experimental environment rather than in real-life clinical settings. The purpose of this study is to compare and contrast the performance of the IOLMaster 500 with the Aladdin using real-life clinical data from an office audit.

The results from this study were presented as a free paper at the European Society of Cataract and Refractive Surgeons (ESCRS) 2021 Virtual Winter Meeting, held from 19th to 21st February 2021.

## Materials and methods

A retrospective analysis of records of patients who presented for cataract surgery at an ophthalmology office in Northern Ontario, Canada between October 2019 and March 2020 was performed. The office is shared by three ophthalmologists and houses both the IOLMaster 500 (v. 7.7) and the Aladdin (v. 1.1.3) biometers. All patients who present for cataract surgery at the office get their measurements on both these biometers. Biometry measurements are performed and recorded by trained ophthalmology technicians during the course of the patient visits. Patients also undergo detailed clinical evaluation by ophthalmologists during their visit, which is recorded in their charts.

Based on the analysis of the patient charts, the data collected for the purpose of the study included patient demographics (age, sex), clinical exam findings (affected eye/s, cataract morphology, and type), and AL measurements recorded by the two biometers (the IOLMaster 500 and the Aladdin). The number of eyes for which AL measurements were successfully obtained by the IOLMaster 500 and the Aladdin was recorded. These were analyzed and compared using the Chi-squared test. For eyes with successful measurements, mean ALs in the two biometer groups were calculated and the unpaired t-test was used for statistical analysis. For both comparisons, a p-value of <0.05 was considered statistically significant. For eyes with unsuccessful measurements, cataract morphology and type were analyzed with a view to determining the possible causes of the biometer's failure to obtain a measurement. The study received a waiver from the institutional Ethics Review Board.

## Results

A total of 197 patients (393 eyes) were included in the study. There were 91 males and 106 females. The mean age of the patients was 71.76 ±8.45 years (range: 40-94 years). Table 1 and Table 2 show the AL measurements recorded by the IOLMaster 500 and the Aladdin.

**Table 1:** Axial length measurements (successful and unsuccessful) by the two biometers

	Axial length by the Aladdin	Axial length by the IOLMaster
Total number of eyes	393	393
Measured/recorded	367	386
Not measured/not recorded	26	7
P-value (Chi-squared test)	0.000727	

**Table 2:** Axial length measurements (mean and standard deviation) by the two biometers

	Axial length (mm) (mean ±standard deviation)
IOLMaster	24.04 ±1.32
Aladdin	24.04 ±1.34
P-value (unpaired t-test)	0.9165

As shown in Table 1, the IOLMaster 500 was unable to obtain measurements of AL in seven eyes and the Aladdin in 26 eyes. The difference was statistically significant (1.8% versus 6.6%, p=0.0007). Table 2 shows the mean values (with standard deviation) for AL between the two biometers for eyes that were successfully measured/recorded. In eyes successfully measured, the mean AL for the IOLMaster was 24.04±1.32 mm, while it was 24.04 ±1.34 mm for the Aladdin. The difference was not statistically significant (p=0.9165).

For eyes in which AL was not successfully measured by either of the two biometers, patient charts were reviewed to find morphology and type of cataract (based on the World Health Organization Simplified Cataract Grading System, 2002) (Table 3). As seen in Table 3, among the eyes with unmeasured AL, a large percentage had advanced or central cataracts.

**Table 3:** Cataract morphology and type among eyes with unsuccessful axial length measurements

Cataract morphology and type	Number of eyes (%)
Nuclear sclerosis grade	
3	2 (7%)
4	7 (25%)
Posterior subcapsular cataract grade	
2	4 (14%)
3	4 (14%)
Anterior subcapsular cataract	
Present	2 (7%)
Mature or total cataract	
Present	4 (25%)

## Discussion

Our study analyzed real-life clinical data from an office audit to compare two different instruments and technologies for optical biometry (the IOLMaster 500 based on PCI versus the Aladdin based on OLCI); to the best of our knowledge, this is one of the largest studies of this nature. 

Accurate and repeatable biometry measurements are of utmost importance for ideal post-cataract surgery refractive outcomes [3,4]. Our study shows that the IOLMaster 500 performed better than the Aladdin in terms of the number of eyes for which measurements of AL were successfully obtained. Thus, IOL power calculation was possible for more eyes with the IOLMaster 500 compared to the Aladdin, in our practice.

Our study also attempted to explore the relationship between unsuccessful measurement of AL by the two biometers and the morphology and type of cataract. Being a retrospective study, a cause-and-effect relationship could not be established. However, eyes with unmeasured AL had a high percentage of advanced, mature/total, and posterior subcapsular cataracts, which were likely responsible for the unsuccessful measurements [11,12]. Our practice serves as a referral center that caters to a large and scattered population in Northern Ontario. This is a region with a vast geographic extent and significant physician shortages, where a lot of patients have to travel long distances for access to cataract surgery [13,14]. Our cataract practice hence deals with a lot of patients with advanced cataracts, which may explain the difference in successful measurements between the two biometers. In contrast to some of the previously published literature [9], we hypothesize that PCI may be better than OLCI in obtaining successful measurements in eyes with advanced and central cataractous changes. As reported previously in the literature [7-10], our study reiterates that AL measurements by the two biometers show no statistically significant difference, as long as the machine successfully measures these parameters.

There are a few limitations to our study. Being a retrospective study, a direct cause-and-effect relationship for unmeasured AL values could not be determined. It is possible that the same technician may not have performed both tests on one patient. Also, since our office had acquired the IOLMaster 500 before the Aladdin, it may be possible that our technicians are more adept at using the IOLMaster. However, the relative ease and rapidity of the Aladdin measurements [8] may make technician training and skills less significant [15].

We are aware from the published literature that both the IOLMaster 500 and the Aladdin have their distinct advantages and disadvantages [7-10]. Hence, a physician’s scope of practice (especially the morphology and type of cataracts the practice commonly encounters) may be the deciding factor regarding the preferred biometer choice.

## Conclusions

For patients undergoing cataract surgery, accurate measurement of AL is of utmost importance for accurate IOL power calculation, so as to achieve optimal postoperative refraction. Our study shows that the IOLMaster 500 and the Aladdin biometers provide comparable AL measurements (as long as the measurements are successfully obtained). This is in agreement with the previously published literature. However, in our study, the IOLMaster 500 was significantly better in terms of the number of eyes for which successful measurements of AL were obtained. Thus, IOL power calculation was possible in more eyes with the IOLMaster 500 compared to the Aladdin, in our study.

One of the factors responsible for this difference may be the high numbers of advanced and central cataracts treated at our practice, possibly due to its geographic location with its associated local population and health demographic. Thus, we conclude that the type of patients and cataracts that a practice caters to may be an important factor in determining the biometer choice. For ophthalmology practices that treat a large number of advanced cataracts and posterior subcapsular cataracts, the IOLMaster 500 (based on PCI) may be better suited than the Aladdin (based on OLCI).
